# Trunk function: the core of mobility performance in wheelchair tennis

**DOI:** 10.3389/fspor.2026.1783088

**Published:** 2026-03-25

**Authors:** Rienk M. A. van der Slikke, Thomas Rietveld, Vicky L. Goosey-Tolfrey, Monique A. M. Berger

**Affiliations:** 1 Centre of Expertise Health Innovation, The Hague University of Applied Sciences, The Hague, Netherlands; 2 Department of Biomechanical Engineering, Delft University of Technology, Delft, Netherlands; 3 Peter Harrison Centre for Disability Sport, School of Sport, Health and Exercise Sciences, Loughborough University, Loughborough, United Kingdom; 4 Department of Human Movement Sciences, Faculty of Behavioural and Movement Sciences, Vrije Universiteit Amsterdam, Amsterdam, Netherlands

**Keywords:** classification, paralympics, sports performance, trunk function, wheelchair athletes

## Abstract

**Introduction:**

Classification in Paralympic sport aims to create a competitive and fair environment by reducing the impact of impairment on the ability to perform sport-specific activities. In wheelchair tennis (WT), current classification criteria largely rely on expert opinion rather than empirical evidence, particularly regarding trunk function. This study investigated the relationship between upper-body strength (arm, trunk) and wheelchair mobility performance in elite WT players to attain evidence-informed classification.

**Methods:**

Fifty-one WT players (men, women, and quad division) were assessed during standardized field tests and match play. Upper-body strength was measured using isometric arm and trunk-related force tests, while mobility performance was quantified using inertial sensors, capturing speed, acceleration, and rotational metrics. Associations between strength and mobility performance outcomes were assessed using Pearson/Spearman correlations. Differences between classification-based trunk function groups (0, 1, 2) were explored using T-tests and effect sizes.

**Results:**

Correlations between strength and mobility performance were modest (r = 0.26–0.62). Push and pull forces showed the highest associations with sprint and rotational performance (push up to r = 0.54; pull up to r = 0.62). Comparisons between trunk function groups revealed substantial differences, particularly in acceleration and rotation, with large effect sizes (ES = 1.18–2.43) between athletes with full vs. minimal trunk control.

**Discussion:**

Trunk function plays a critical role in WT mobility and is underrepresented in the current classification system. Future classification systems should include functional measures that reflect the impact of impairment on sport-specific activities. Particular attention should be given to dynamic trunk movements during acceleration and rotation to ensure a more evidence-based and functionally relevant approach.

## 1 Introduction

Wheelchair tennis (WT) is one of the fastest growing wheelchair-based Para sports, fully integrated into all major Grand Slam events. The game closely resembles able-bodied tennis but includes adaptations such as wheelchair use and the allowance of a second bounce. WT performance results from the complex interaction between athlete, wheelchair, and environment (1, 2), and is characterized by frequent directional changes and short, high-intensity sprint bouts (3). Upper-body function is essential for balance, propulsion, speed, and manoeuvrability, as well as for sport-specific actions such as stroke execution and serving (4). Despite its apparent importance, the relationship between upper-body function and wheelchair mobility performance has not been systematically investigated.

Classification in wheelchair sports aims to minimize the impact of impairment on competition outcomes, ensuring success depends on skill, training and strategy rather than the degree of impairment (5). It operates as a selective system that groups athletes by impairment type and severity based on its effect on propulsion, manoeuvrability, balance, stroke execution and racket control. The IPC Classification Code (6) mandates evidence-based classification using objective measures and a clear link to performance. In WT, classification is divided in two classes: the “Open” division, separated by sex, including players with permanent lower-limb impairments, and the “Quad” division, which combines men and women with additional upper-limb restrictions (7). Classification currently allocates up to 14 points based on arm and trunk function: each arm is scored 0–4 points (with the dominant arm weighted twice), while trunk function contributes only 0–2 points. Athletes scoring 10 points or less are eligible for the “Quad” division. These thresholds and weighting factors are based on expert opinion rather than empirical evidence.

Eligible players must have a permanent physical impairment that significantly limits running and change of direction, due to lower-limb involvement (Open division) or combined lower- and upper-limb impairment affecting racket handling and wheelchair propulsion (Quad division) (7). Previous research has explored minimum impairment criteria and identified class differences in serving and mobility performance (8–11). The extent to which trunk and arm function influence mobility performance, and thus classification fairness, has not been systematically examined. Moreover, racket use, an integral part of the sport, is not considered in classification, even though it may affect mobility differently for athletes with varying arm and hand function (12). Strength assessments are commonly used in other Para sports for classification, with standardized protocols being recommended (13–15). Evidence from wheelchair rugby suggests that arm-trunk strength can distinguish impairment levels (10, 11, 16, 17), only this area remains unexplored in WT. The current study therefore focuses on investigating the relationship between trunk and arm function and wheelchair mobility performance in elite WT players.

Wheelchair mobility performance can be evaluated both during match play (3, 10, 11, 18) and through standardized field tests (19). Technological advancements, such as inertial measurement units (IMUs), enable detailed and objective quantification of speed, acceleration, and rotational movements, providing data that are highly relevant for classification (20–22). Compared to other measurement systems, IMUs are particularly suitable for field-based performance evaluation because they directly capture accelerations, making them highly sensitive to detecting changes in force production — a key aspect in studies examining athlete strength and propulsion mechanics. Moreover, IMUs allow the use of the same measurement method during both controlled field tests and real match play, ensuring that identical performance variables can be compared across testing environments and competitive settings. WT presents unique demands compared to other wheelchair sports due to its high rotational requirements and rapid transitions. Performance also varies by division and sex, with Quad players typically showing lower speeds than Open players, and men and women showing distinctive speed and rotational characteristics (2, 3, 10, 11). These insights highlight the need for precise measurement methods to capture mobility performance under realistic tennis conditions, forming the basis for the current study.

The aim of this study was to determine the relationship between trunk and arm strength and wheelchair mobility performance in elite WT players, and to evaluate its implications for classification. Mobility performance was selected because it reflects the athlete's ability to execute essential tennis movements, providing a direct link between impairment and functional capacity. By focusing on this core aspect, the study offers a controlled and objective way to evaluate functional impact. To achieve this, standardized strength assessments were combined with on-court mobility tests and match play analysis. In addition, classification-relevant factors such as racket use (tested with and without a racket) and side dominance (dominant vs. non-dominant arm) were explored to better understand their influence on mobility performance.

## 2 Methods

### 2.1 Participants

Fifty-one international WT players participated in this study, divided into three divisions: Men's (*n* = 24), Women's (*n* = 12), and Quad (*n* = 15; all male). No female athletes were included in the Quad division, which reflects the current competitive landscape where the number of active female Quad players is extremely limited. Athletes were included based on participation in major international tournaments during the 2023 season, ensuring a sample of elite-level players representative of current classification practice. All participants were free from acute injuries and competed in their regular sports wheelchair. The study was approved by the Ethics Committee of The Hague University of Applied Sciences (“*The muscle power impairment—mobility performance relationship in elite wheelchair tennis*”), all participants were informed about the study's purpose and provided written informed consent prior to participation.

#### 2.1.1 Sample size

A formal *a priori* sample size calculation was not feasible due to the absence of directly comparable effect sizes for combined assessments of trunk function, arm strength and IMU-based wheelchair mobility performance. In line with recommendations for sample-size justification in studies involving small, highly specialised populations, we therefore adopted a resource-based sampling approach (23). The final sample reflects the maximum number of eligible elite wheelchair tennis players available during the international tournaments in which data collection occurred. Given this fixed sample size, the study is adequately powered to detect medium-to-large effects—effect sizes commonly reported in wheelchair mobility and propulsion research.

#### 2.1.2 Trunk scores

Athletes were categorized into trunk function groups based on the International Tennis Federation classification system (7). Trunk score 0: Athletes with non-functional trunk (*n* = 8; all SCI); Trunk score 1: Athletes with fair to good trunk control (*n* = 10; 9 SCI, 1 neurological impairment); and Trunk score 2: Athletes with full trunk control (*n* = 33; 8 SCI, 8 amputations, 8 congenital limb deficiencies, 5 neurological impairments, 2 cerebral palsy, 2 orthopaedic conditions). A complete overview of primary impairments is provided in Supplementary Table S1. Grouping by trunk score allowed comparison of functional differences aligned with current classification criteria. Trunk function categorization for Quad players was determined by certified ITF classifiers, whereas Open-division athletes were assigned trunk scores by the research team in accordance with ITF guidelines; all classifications were independently verified by a medical member of the ITF research team to ensure accuracy and consistency.

### 2.2 Protocol

All measurements were carried out on hard-court tennis surfaces across the three tournament locations. Testing at the ABN AMRO Open (Rotterdam) and the British Open (Nottingham) took place indoors, while testing at the BNP World Team Cup (Vilamoura) occurred outdoors under stable, calm and sunny conditions. A short, self-selected warm-up was required before the on-court field tests, and athletes followed their usual pre-match warm-up routine before match-play measurements. Prior to each test component, the procedure was explained and demonstrated at low intensity by the test leader to ensure adequate familiarization.

Testing sessions were scheduled in coordination with the tournament programme. Whenever possible, field tests were conducted on days without matches; if this was not feasible, testing was never performed after a match to minimise the influence of fatigue. Athletes used their own sports wheelchairs with their usual configuration and strapping setup, identical to match conditions. Importantly, all assessments formed part of an independent research protocol and had no influence on the athletes’ classification status. This was explicitly communicated to all participants to ensure that they felt free to perform maximally.

#### 2.2.1 Isometric strength measures

Force was measured with a Mecmesin Advanced Force Gauge (AFG) and a 1,000 N S-Beam load cell (Harttech, Netherlands), connected to a laptop running a custom MATLAB script that recorded data at 20 Hz and provided audio start and stop cues.

**Figure 1:**
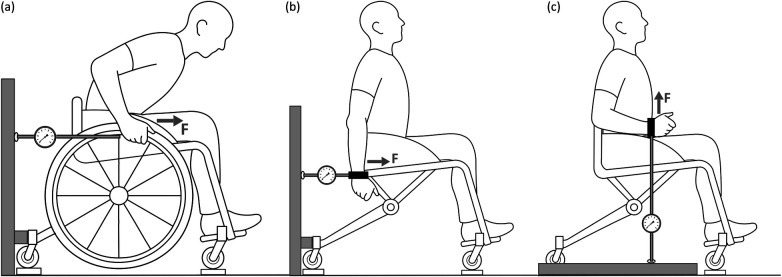
Schematic representation of the measurement frame with the force transducer and different measurement positions and force directions (F). **(a)** Push on the rim, with the strap fixed to the top of the rim. **(b)** Push anteflexion full arm (in a similar way for the upper arm). **(c)** Push flexion of the forearm. All measurements were **also performed in opposite force direction** (for extension and pull). During the measurements the main wheels are lifted from the floor or removed.

To minimize participant burden and ensure sport-specific relevance, all strength measurements were conducted with athletes seated in their own sports wheelchairs. The wheelchair frame was braced against the frame of the measurement setup (Figure 1), while a force transducer attached via a tension strap allowed free force direction. For push and pull measurements, the wheelchair wheels were slightly lifted, with straps securing the rim to enable natural force application in a static position. The non-measured side was strapped for stability, and the demountable measurement frame ensured easy transport. All strength assessments were performed in the athletes' own sports wheelchairs using their standard competition configuration, including individual frame setup and strapping arrangements; no additional trunk support was provided beyond what athletes routinely use in competition, and trunk straps were only used by those who normally compete with them.

The force measurements included isolated arm force assessments for the upper arm, full arm, and forearm (in 90° flexion), in both extension and flexion directions. Additionally, push and pull forces on the wheelchair rim were measured as multi-joint tests on both sides (see Supplementary Material, Table S2). Each measurement lasted 5 s, comprising a 2 s build-up phase followed by 3 s of sustained maximal effort. Within the 3 s maximal phase, a sliding 1-second window was applied to the force–time signal to identify the most stable high-force segment. The outcome measure was defined as the mean force within this highest 1 s window, which reduces the impact of short fluctuations and ensures that the reported value reflects a sustained maximal output rather than a transient peak. This procedure was done twice for each force registration, with the mean of the peak forces used for further analysis.

#### 2.2.2 Wheelchair mobility performance measurement

Wheelchair mobility performance was assessed using a previously validated method (18, 24), employing two synchronised (<0.01 s) xIMU3 (x-IO Technologies) sensors. One sensor was positioned on the right wheel axle, and the other on the centre of the wheelchair frame. During the on-court field tests, an additional IMU was attached to the athlete's trunk (approximately C7) using a vest, to determine trunk angle and trunk acceleration. The IMU-based method for assessing wheelchair mobility performance enabled calculation of key metrics: maximal speed [m/s] (average of the best five sprints during match play); average absolute forward acceleration [m/s^2^] (as a measure of intensity); maximal rotational speed [^o^/s] (average of the best five rotations during match play); average rotational acceleration [^o^/s^2^] (as a measure of rotational intensity); maximal acceleration per push [m/s^2^] (average of the best five sprints during match play); time to cover a specific distance [s] (for on-court tests only); average trunk angle [^o^] (for on-court tests only); backward acceleration trunk relative to chair per push [^o^/s^2^].

#### 2.2.3 Standardized on-court field test

**Figure 2:**
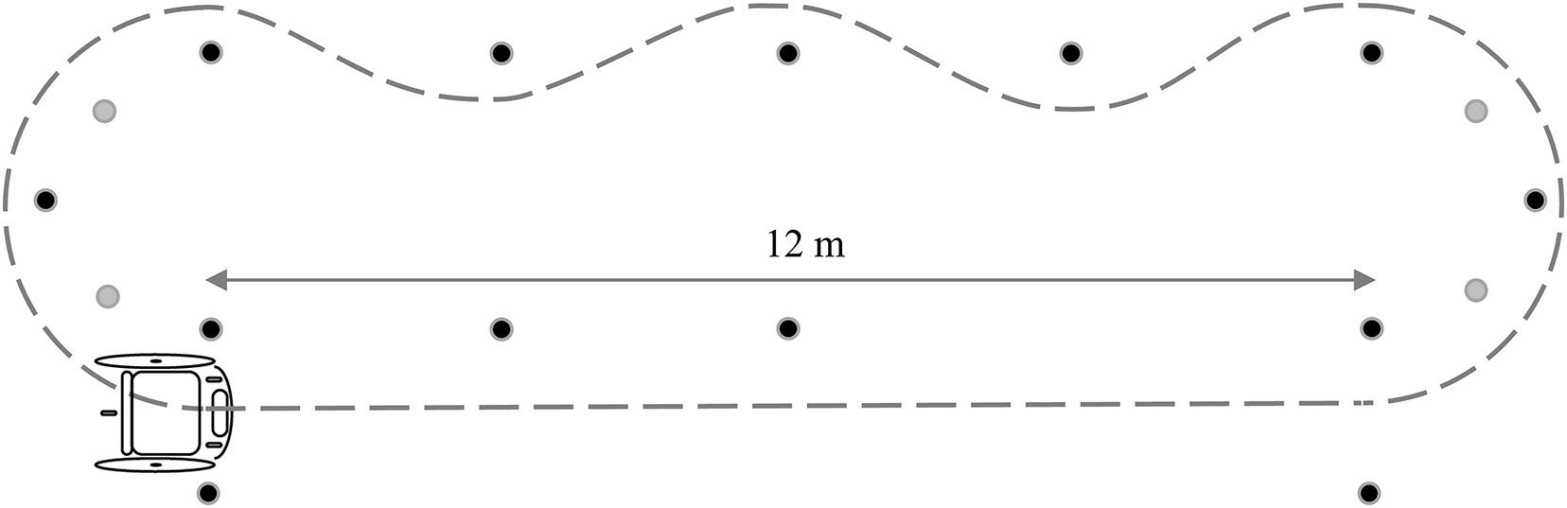
Layout of the test track (without 20 m and spider test), adopted from (21).

The standardized on-court test consisted of a selection of sport-specific tests (21), with the addition of a 20-metre sprint. Each test was performed both with and without a racket to assess the impact of racket use on wheelchair mobility performance. The tests included (1) 20-metre sprint, (2) 12-metre sprint to full stop, (3) 12-metre intermittent sprint, with a stop-and-go at 3 and 6 metre and a full stop at 12 metres, (4) Sprint-curve-slalom-curve, with 12 metre sprint, a 6 metre curve, a 12 metre slalom with cones at 3–6–9 metre and a final 6 metre curve, (5) 180 turn on the spot (left and right) (Figure 2).

#### 2.2.4 On-court match play

Wheelchair mobility performance was measured for each athlete during one full match in the respective tournament using the IMU sensors. All match-play assessments were conducted during major international tournaments, ensuring a competitive context in which athletes performed at maximal effort; tournament phase and ranking were not analysed.

### 2.3 Statistical analysis

All statistical analyses were performed using SPSS 28, with significance set at *p* < 0.05. Normality was assessed prior to correlation analyses (25). Normality of all strength variables (push and pull forces) and all wheelchair-mobility outcomes (speed, acceleration, rotational speed/acceleration, trunk kinematics) was assessed using the Shapiro–Wilk test. If assumptions were met, Pearson correlation coefficients were calculated; Spearman's rank correlation was applied as a non-parametric alternative. Correlations were interpreted as weak (0.10–0.39), moderate (0.40–0.69), strong (0.70–0.89), and very strong (0.90–1.00) (26).

Correlation coefficients were computed to examine relationships between upper-body strength outcomes (push and pull forces) and wheelchair mobility performance metrics obtained from standardized on-court tests and match play. To address multiplicity in correlation analyses testing the same hypothesis, we controlled the false discovery rate using the Benjamini–Hochberg procedure within each test block (Match, 20 m, 12 m, 3-3-6 m, Sprint-curve-slalom, Turn R, Turn L). We report FDR-adjusted p-values as q; correlations were considered significant at q < .05. Differences in wheelchair mobility performance between trunk function groups (Trunk 0–2, 0–1, and 1–2) were further analysed using Mann–Whitney *U*-tests, due to a relatively small sample size. Because three pairwise comparisons were performed, *p*-values were Bonferroni-corrected (padj = *p* × 3). Adjusted *p*-values are reported in the tables. To quantify the magnitude of the differences, Cohen's *d* effect sizes were calculated (27). Effect sizes (ES) were interpreted as small (0.2), medium (0.5), and large (≥0.8).

In addition, exploratory tests were conducted to address classification-relevant factors for upper body strength. To examine the relationship between dominant and non-dominant arm strength, correlations were calculated for all strength tests on both sides (upper-arm flexion/extension, full-arm flexion/extension, forearm flexion/extension, and push/pull force). Dominance was defined according to the athletes' self-reported playing arm. Paired-samples *t*-tests were used to compare wheelchair mobility performance outcomes measured with and without a racket during standardized field tests.

## 3 Results

### 3.1 Strength and wheelchair mobility performance

Correlations between upper-body strength and wheelchair mobility performance revealed distinct patterns across contexts. Standardized on-court field tests demonstrated stronger relationships: pull force displayed the highest correlations with sprint times and rotational speeds, particularly in the 20 m sprint (r = 0.60), 12 m sprint (r = 0.57), and left/right rotations (r = 0.62 and r = 0.58). For example, maximal pull force was substantially higher in athletes with full trunk function (Trunk 2: 274 ± 113 N) compared to those with no trunk function (Trunk 0: 173 ± 64 N). Correlations between push/pull forces and trunk motion outcomes were weak or inconsistent. Between match play mobility performance and push/pull forces, correlations were generally weak, although pull force showed slightly stronger associations with average acceleration (r = 0.29–0.43; Table 1).

**Table 1:** Pearson correlations (r) between push/pull force (N) and wheelchair mobility performance; with significance (p) shown and significance after FDR correction (q, Benjamini-Hochberg).

		Push			Pull		
Match or part	Performance outcome	r	*p*	q BH	r	*p*	q BH
Match	Maximal speed (best 5) [m/s]	**0**.**33**	0.01798	**0**.**02568**	**0**.**32**	0.02302	**0**.**02877**
	Average acceleration [m/s^2^]	**0**.**29**	0.03812	**0**.**03812**	**0**.**43**	0.00157	**0**.**01569**
	Maximal rotational speed (best 5) [^o^/s]	**0**.**30**	0.03118	**0**.**03465**	**0**.**36**	0.01033	**0**.**01807**
	Average rotational speed [^o^/s]	**0**.**35**	0.01084	**0**.**01807**	**0**.**36**	0.00969	**0**.**01807**
	Acceleration push (>mean, best 5)	**0**.**37**	0.00789	**0**.**01807**	**0**.**40**	0.00399	**0**.**01807**
20 m sprint	Maximal speed [m/s]	**0**.**34**	0.01473	**0**.**02456**	**0**.**40**	0.00339	**0**.**00847**
	Acceleration push (>mean) [m/s^2^]	**0**.**39**	0.00468	**0**.**00935**	**0**.**50**	0.00025	**0**.**00083**
	Time 20 m [s]	**0**.**54**	0.00008	**0**.**00039**	**0**.**60**	0.00001	**0**.**00008**
	Average trunk angle [^o^]	0.05	0.73347	0.73347	0.07	0.62776	0.72474
	Acceleration backward [m/s^2^]	0.07	0.65227	0.72474	0.24	0.10230	0.14615
12 m sprint	Max speed [m/s]	**0**.**53**	0.00006	**0**.**00020**	**0**.**61**	0.00000	**0**.**00002**
	Acceleration push (>mean) [m/s^2^]	**0**.**38**	0.00532	**0**.**00887**	**0**.**44**	0.00111	**0**.**00221**
	Time 10 m [s]	**0**.**50**	0.00019	**0**.**00048**	**0**.**57**	0.00002	**0**.**00009**
	Average trunk angle [^o^]	0.23	0.12562	0.13958	**0**.**34**	0.02298	**0**.**03283**
	Acceleration backward [m/s^2^]	0.15	0.30009	0.30009	0.29	0.04817	0.06022
3–3–6 m interval	Maximal speed [m/s]	**0**.**54**	0.00005	**0**.**00017**	**0**.**57**	0.00001	**0**.**00009**
	Acceleration push (>mean) [m/s^2^]	**0**.**45**	0.00095	**0**.**00159**	**0**.**47**	0.00056	**0**.**00113**
	Time 10 m [s]	**0**.**48**	0.00044	**0**.**00109**	**0**.**57**	0.00002	**0**.**00009**
	Average trunk angle [^o^]	0.14	0.35401	0.35401	0.26	0.08935	0.11168
	Acceleration backward [m/s^2^]	0.20	0.17596	0.19551	0.27	0.06363	0.09090
Sprint-curve-slalom	Maximal speed [m/s]	**0**.**47**	0.00048	**0**.**00114**	**0**.**52**	0.00009	**0**.**00054**
	Average rotational speed [^o^/s]	**0**.**46**	0.00059	**0**.**00117**	**0**.**56**	0.00002	**0**.**00024**
	Acceleration push (>mean) [m/s^2^]	**0**.**40**	0.00366	**0**.**00488**	**0**.**47**	0.00045	**0**.**00114**
	Average rotational acceleration [^o^/s^2^]	**0**.**46**	0.00068	**0**.**00117**	**0**.**48**	0.00037	**0**.**00114**
	Average trunk angle [^o^]	0.15	0.33912	0.33912	0.31	0.04312	0.05175
	Acceleration backward [m/s^2^]	0.28	0.05720	0.06240	**0**.**43**	0.00267	**0**.**00400**
Turn R	Maximal rotational speed [^o^/s]	**0**.**47**	0.00050	**0**.**00075**	**0**.**61**	0.00001	**0**.**00001**
	Average rotational acceleration [^o^/s^2^]	**0**.**48**	0.00032	**0**.**00065**	**0**.**58**	0.00001	**0**.**00002**
	Average trunk angle [^o^]	0.00	0.99436	0.99436	0.19	0.20327	0.24393
Turn L	Max rotational speed [^o^/s]	**0**.**54**	0.00005	**0**.**00015**	**0**.**62**	0.00001	**0**.**00001**
	Average rotational acceleration [^o^/s^2^]	**0**.**47**	0.00048	**0**.**00073**	**0**.**49**	0.00024	**0**.**00047**
	Average trunk angle [^o^]	0.09	0.56365	0.56365	0.19	0.21078	0.25294

Values significant after FDR correction (q BH < .05) are shown bold with grey background.

Group comparisons indicated significant differences in wheelchair mobility performance across trunk function groups (Table 2), particularly in metrics related to speed, acceleration, and rotational movement. Players in the full trunk function group (Trunk 2) consistently achieved higher maximal speeds during both match play and across sprint tests, with the largest effect sizes observed in the 20 m and 12 m sprints (*p* < 0.01, ES > 1). During match play, athletes with full trunk control reached markedly higher maximal speeds (4.1 ± 0.4 m/s) than athletes with non-functional trunk (3.2 ± 0.5 m/s). Acceleration during the push phases was also greater in this group, particularly in the sprint and slalom tests (*p* < 0.01, ES > 2.3).

**Table 2:** Differences in trunk group scores for force, match wheelchair mobility performance and field-test wheelchair mobility performance separated by trunk group (0, 1, 2).

		0	1	2	0–2		0–1		1–2	
Match or test	Perfromance outcome	Mean ± SD	Mean ± SD	Mean ± SD	P_adj_ MW	ES (‘d)	P_adj_ MW	ES (‘d)	P_adj_ MW	ES (‘d)
Force	Max push [N]	195 ± 55	238 ± 97	245 ± 105	0.7900	−0.51	0.8590	−0.53	1.0000	
	Max pull [N]	173 ± 64	266 ± 99	274 ± 113	**0**.**0339**	**−0**.**95**	0.0989	**−1**.**08**	1.0000	
Match	Max speed (B5) [m/s]	3.2 ± 0.5	3.5 ± 0.5	4.1 ± 0.4	**0**.**0015**	**−1**.**98**	0.7442	−0.56	**0**.**0092**	**−1**.**35**
	Avg acceleration [m/s^2^]	0.78 ± 0.05	0.99 ± 0.19	1 ± 0.17	**0**.**0038**	**−1**.**41**	**0**.**0299**	**−1**.**44**	1.0000	
	Max rot. speed (B5) [^o^/s]	289 ± 12	337 ± 44	363 ± 54	**0**.**0003**	**−1**.**5**	**0**.**0493**	**−1**.**43**	0.8624	−0.5
	Avg rotational speed [^o^/s]	83 ± 10	105 ± 19	111 ± 19	**0**.**0009**	**−1**.**54**	**0**.**0299**	**−1**.**36**	0.8239	−0.32
	Acc push (>mean, B5) [m/s^2^]	2.6 ± 0.9	3 ± 0.8	4.1 ± 0.9	**0**.**0053**	**−1**.**66**	0.9851	−0.48	**0**.**0121**	**−1**.**24**
Sprint 20 m	Max speed [m/s]	3.8 ± 0.4	3.8 ± 1.4	4.7 ± 0.5	**0**.**0011**	**−1**.**71**	1.0000		0.1144	**−1**.**04**
	Acc push (>mean) [m/s^2^]	2.2 ± 0.6	3.3 ± 1	4.6 ± 1.1	**0**.**0001**	**−2**.**39**	0.0790	**−1**.**25**	**0**.**0084**	**−1**.**24**
	Time 20 m [s]	7.4 ± 0.7	6.7 ± 1	6.3 ± 0.7	**0**.**0034**	**1**.**54**	0.2498	0.71	0.6549	0.6
	Avg trunk angle [^o^]	51.6 ± 12.8	49.6 ± 19.5	29.9 ± 12.8	**0**.**0025**	**1**.**7**	1.0000		**0**.**0117**	**1**.**38**
	Acc backward [m/s^2^]	−9.8 ± 2.9	−12 ± 4.2	−16.7 ± 5.7	**0**.**0022**	**1**.**3**	0.5338	0.59	0.1016	**0**.**87**
Sprint 12 m	Max speed [m/s]	3.3 ± 0.4	3.6 ± 0.5	4.1 ± 0.4	**0**.**0015**	**−1**.**9**	0.8492	−0.6	**0**.**0117**	**−1**.**23**
	Acc push (>mean) [m/s^2^]	2.1 ± 0.8	2.9 ± 0.9	4.3 ± 1	**0**.**0001**	**−2**.**37**	0.3500	**−0**.**87**	**0**.**0008**	**−1**.**52**
	Time 10 m [s]	4.5 ± 0.4	4.2 ± 0.5	3.8 ± 0.3	**0**.**0012**	**1**.**96**	0.9851	0.5	**0**.**0315**	**1**.**24**
	Avg trunk angle [^o^]	54.1 ± 11.6	37.6 ± 6.2	25.3 ± 12.7	**0**.**0014**	**2**.**29**	**0**.**0425**	**1**.**87**	**0**.**0145**	**1**.**04**
	Acc backward [m/s^2^]	−9.4 ± 3.9	−12.6 ± 4.6	−16.7 ± 4.1	**0**.**0015**	**1**.**77**	0.3292	0.73	0.0537	**0**.**96**
Sprint Interval	Max speed [m/s]	2.6 ± 0.4	2.9 ± 0.4	3.4 ± 0.3	**0**.**0010**	**−2**.**02**	0.6465	−0.75	**0**.**0127**	**−1**.**13**
	Acc push (>mean) [m/s^2^]	1.9 ± 0.8	2.5 ± 0.9	3.8 ± 0.8	**0**.**0002**	**−2**.**43**	0.4812	−0.76	**0**.**0011**	**−1**.**57**
	Time 10 m [s]	7.5 ± 1.1	6.4 ± 0.8	6.1 ± 0.8	**0**.**0050**	**1**.**71**	0.2078	**1**.**16**	0.7103	0.48
	Avg trunk angle [^o^]	54.4 ± 13	43 ± 10.4	32.7 ± 13	**0**.**0031**	**1**.**66**	0.3456	**0**.**97**	**0**.**0494**	**0**.**82**
	Acc backward [m/s^2^]	−8.2 ± 3.8	−9.7 ± 3.3	−13.2 ± 2.9	**0**.**0052**	**1**.**61**	1.0000	0.41	**0**.**0248**	**1**.**19**
Sprint slalom	Max speed [m/s]	3.4 ± 0.4	3.7 ± 0.5	4.2 ± 0.4	**0**.**0005**	**−1**.**88**	0.9653	−0.52	**0**.**0313**	**−1**.**17**
	Avg rotational speed [^o^/s]	39.2 ± 4.9	44.2 ± 5.7	49.9 ± 4.9	**0**.**0004**	**−2**.**19**	0.0774	**−0**.**93**	**0**.**0107**	**−1**.**12**
	Acc push (>mean) [m/s^2^]	2.1 ± 0.8	3 ± 1	4.2 ± 0.9	**0**.**0002**	**−2**.**36**	0.2078	**−0**.**87**	**0**.**0043**	**−1**.**34**
	Avg rotational acc [^o^/s^2^]	191 ± 59	224 ± 87	290 ± 89	**0**.**0140**	**−1**.**18**	1.0000	−0.44	0.0815	−0.75
	Avg trunk angle [^o^]	59.8 ± 10.8	45.1 ± 6.3	32.9 ± 11.8	**0**.**0004**	**2**.**32**	**0**.**0264**	**1**.**66**	**0**.**0102**	**1**.**11**
	Acc backward [m/s^2^]	−7.2 ± 3.5	−10 ± 2.5	−12.1 ± 2.8	**0**.**0055**	**1**.**67**	0.3292	**0**.**93**	0.1270	0.78
Turn R	Max rotational speed [^o^/s]	285 ± 45	348 ± 60	411 ± 61	**0**.**0003**	**−2**.**15**	0.0954	**−1**.**16**	**0**.**0267**	**−1**.**03**
	Avg rotational acc [^o^/s^2^]	390 ± 144	526 ± 119	705 ± 163	**0**.**0005**	**−1**.**97**	0.1170	**−1**.**05**	**0**.**0048**	**−1**.**16**
	Avg trunk angle [^o^]	69.1 ± 11.9	57.3 ± 4.8	46.3 ± 9.6	**0**.**0004**	**2**.**27**	0.1028	**1**.**33**	**0**.**0033**	**1**.**25**
Turn L	Max rotational speed [^o^/s]	284 ± 56	334 ± 59	400 ± 67	**0**.**0011**	**−1**.**79**	0.2487	**−0**.**87**	**0**.**0178**	**−1**.**01**
	Avg rotational acc [^o^/s^2^]	347 ± 143	448 ± 108	608 ± 159	**0**.**0019**	**−1**.**67**	0.2959	**−0**.**82**	**0**.**0075**	**−1**.**07**
	Avg trunk angle [^o^]	68.4 ± 13.8	55.1 ± 6.9	46.5 ± 10.2	**0**.**0009**	**1**.**99**	0.0807	**1**.**24**	0.0538	**0**.**9**

Significance level of the Mann–Whitney test (P MW), with *p* < 0.05 marked bold and grey and the effect size (ES), with ES > 0.8 marked bold and grey. Maximal, max; Average, avg; Acceleration, acc; Best 5, B5.

Rotational speed and rotational acceleration also varied between groups, with the largest differences observed in the turn and slalom tests, where Trunk 2 exhibited higher values than Trunk 0 (*p* < 0.01, ES > 1). Mean trunk angle was consistently lower (indicating greater forward flexion) in the Trunk 2 group, particularly in the 12 m sprint, slalom, and turn tests (*p* < 0.01, ES > 1.5).

During match play, maximum speed and push acceleration mainly differed between Trunk 1 and 2, whereas average acceleration and rotational components showed the largest differences between Trunk 0 and 1. A similar pattern emerged in the field test data, with the largest effect sizes in forward sprints observed between Trunk 1 and 2 (greater than between Trunk 0 and 1). In the 20-m sprint, Trunk 2 athletes achieved higher maximal speed (4.7 ± 0.5 m/s) and peak acceleration (4.6 ± 1.1 m/s²) than Trunk 0 athletes (3.8 ± 0.4 m/s; 2.2 ± 0.6 m/s²). For rotational components (slalom and turns), the differences in effect size magnitude between Trunk 1 and 2 were smaller compared to those between Trunk 0 and 1.

### 3.2 Arm strength side and racket use

Arm strength measures were moderately to strongly correlated with each other (r = 0.55–0.93; Supplementary Material Table S3). The correlations between sides were high for upper-arm flexion (r = 0.93) and extension (r = 0.92), forearm flexion (r = 0.75) and extension (r = 0.82), as well as push (r = 0.86) and pull (r = 0.83). Performance outcomes with and without a racket were strongly correlated (Table 3; r = 0.73–0.96), suggesting consistent movement patterns. However, significant differences were observed: maximal speed was lower with a racket (*p* < 0.001, ES = −0.63), with sprint times being longer (20 m sprint: *p* < 0.001, ES = 1.02; 10 m sprint: *p* < 0.001, ES = 1.61). Trunk angle was higher when using a racket (*p* < 0.001, ES = 0.64).

**Table 3:** Wheelchair mobility performance with and without racket in the on-court standardized tests, mean difference of all significant differences and correlations.

Test	Perfromance outcome	With racket	No racket	Difference	*p*	Cohen's d	Correlation
20 m sprint	Max speed [m/s]	4.36	4.73	−0.36	**0** **.** **00**	−0.63	*0*.*73*
	Acc push (>mean) [m/s^2^]	4.04	3.95	0.09	0.14	0.15	**0**.**90**
	Time 20 m [s]	6.52	6.14	0.38	**0**.**00**	1.02	**0**.**90**
	Avg trunk angle [^o^]	36.9	30.9	6.0	**0**.**00**	0.64	*0*.*84*
	Acc backward [m/s^2^]	−14.79	−14.73	−0.06	0.45	−0.02	*0*.*76*
12 m sprint	Max speed [m/s]	3.89	4.10	−0.21	**0**.**00**	−1.35	**0**.**96**
	Acc push (>mean) [m/s^2^]	3.73	3.81	−0.08	0.09	−0.18	**0**.**94**
	Time 10 m [s]	3.95	3.73	0.22	0.00	1.61	**0**.**96**
	Avg trunk angle [^o^]	31.1	28.7	2.4	**0**.**00**	0.42	**0**.**93**
	Acc backward [m/s^2^]	−14.80	−15.43	0.63	**0**.**02**	0.27	*0*.*88*
3–3–6 m interval	Max speed [m/s]	3.17	3.37	−0.20	**0**.**00**	−1.21	**0**.**93**
	Acc push (>mean) [m/s^2^]	3.30	3.48	−0.17	**0**.**00**	−0.39	**0**.**92**
	Time 10 m [s]	6.36	6.25	0.11	0.09	0.19	*0*.*80*
	Avg trunk angle [^o^]	37.9	35.9	2.0	**0**.**01**	0.37	**0**.**93**
	Acc backward [m/s^2^]	−11.83	−12.81	0.98	**0**.**00**	0.44	*0*.*82*
Sprint-curve-slalom	Max speed [m/s]	3.96	4.20	−0.24	**0**.**00**	−1.13	**0**.**93**
	Avg rotational speed [^o^/s]	47.2	48.4	−1.2	**0**.**00**	−0.38	*0*.*86*
	Acc push (>mean) [m/s^2^]	3.72	3.71	0.01	0.46	0.01	**0**.**95**
	Avg rotational acc [^o^/s^2^]	260.9	257.1	3.8	0.17	0.13	**0**.**94**
	Avg trunk angle [^o^]	39.5	37.7	1.8	**0**.**02**	0.31	**0**.**93**
	Acc backward [m/s^2^]	−11.14	−11.34	0.20	0.21	0.11	*0*.*85*
Turn R	Max rotational speed [^o^/s]	376.7	387.4	−10.7	**0**.**01**	−0.35	**0**.**91**
	Avg rotational acc [^o^/s^2^]	616.3	655.0	−38.7	**0**.**01**	−0.33	*0*.*83*
	Avg trunk angle [^o^]	52.7	50.9	1.8	**0**.**00**	0.41	**0**.**94**
Turn L	Max rotational speed [^o^/s]	367.8	385.7	−17.9	**0**.**00**	−0.53	*0*.*89*
	Avg rotational acc [^o^/s^2^]	537.6	602.2	−64.6	**0**.**00**	−0.57	*0*.*79*
	Avg trunk angle [^o^]	52.6	51.5	1.0	0.08	0.20	**0**.**92**

Significance level of the paired samples *T*-Test *p* < 0.05 marked bold and grey and correlations over 0.9 marked bold and grey. Maximal, max; Average, avg; Acceleration, acc; Best 5, B5.

## 4 Discussion

This study demonstrates that trunk function, as defined by the ITF classification framework, has a clear and measurable impact on WT mobility performance, particularly in tasks involving rotation and acceleration. Importantly, the present results show that the classification-based trunk groups (0 = no functional trunk control, 1 = partial control, 2 = full control) align with clear, functionally meaningful differences in mobility performance. These findings support the use and potential refinement of trunk-group distinctions within the existing classification framework. Large effect sizes between trunk function groups confirm its relevance for performance differences and provide important insights for classification development. Trunk function should be given greater weight in classification systems, as its impact on mobility performance exceeds that of arm strength. It is important to interpret these findings within the purpose of Paralympic classification, which is not to predict overall performance but to minimise the impact of impairment on the outcome of competition. The observation that trunk function produces systematic and meaningful differences in wheelchair mobility supports the relevance of weighting trunk impairment more strongly within this framework.

The relationship between strength and mobility performance was weaker than anticipated. Correlations between force and match play mobility performance ranged from 0.29 to 0.43, while correlations between force and on-court test mobility ranged from 0.26 to 0.62. This indicates that muscle force only partly explains the variance in mobility performance. These findings align with previous work in wheelchair rugby, where trunk strength was shown to be critical for propulsion and manoeuvrability (16, 28), although the rotational and directional demands in wheelchair tennis differ substantially from rugby, making trunk control particularly relevant for rapid turns and slalom movements. It also confirms that strength alone cannot fully predict WT performance, which is inherently complex and influenced by technical and tactical factors such as racket handling, ball control, and serving (12, 29).

The importance of trunk control for various aspects of WT has been described in the literature (3, 30, 31), but this was the first study linking trunk to wheelchair mobility performance. Effect sizes revealed trivial to small differences between trunk function groups for push force, which might be explained by limited trunk stabilization when athletes are supported by the backrest. In contrast, moderate to large differences were observed for pull force and wheelchair mobility outcomes between Trunk 0 and 2, indicating that the absence of trunk function significantly impacts performance. A methodological consideration is that all strength assessments were conducted in the athletes’ own sports wheelchair configuration to maximise sport-specific relevance; while this enhances ecological validity, the use of individualized strapping and seating may have influenced the absolute force values obtained, particularly for athletes with reduced trunk control.

On-court tests showed consistent and substantial differences between groups, particularly in sprint and rotational tasks, while match play data confirmed similar trends. Maximal speed and push acceleration differed primarily between Trunk 2 and 1, whereas rotational metrics such as average acceleration and rotational speed demonstrated the greatest differences between Trunk 0 and 1. These findings highlight that while full trunk function enhances forward sprint performance, the distinction between minimal and reduced trunk function becomes most pronounced in rotational movements. These differences are critical for WT performance, as rotational aspect plays a key role in WT (2, 3). The classification systems should not only distinguish between full and partial trunk function, but potentially consider an additional partial trunk group, similar to wheelchair rugby (16).

Exploratory analyses revealed additional factors relevant to classification. Racket use significantly affected mobility outcomes during on-court tests, reducing maximal speed and increasing sprint times. This aligns with previous research showing that the use of a tennis racket negatively affects sprint performance and propulsion technique (12, 29). Although movement patterns were highly correlated across conditions, the absolute differences highlight the need to account for racket use in classification research, as previously described by (32) and (33). Investigating the impact of racket use across various classification levels could provide additional insights, with the Quad class players having additional restrictions to their playing arms (34). Similarly, dominant arm strength was consistently higher than non-dominant strength, yet strong correlations between sides suggest that overall strength capacity drives performance more than asymmetry. This finding is especially relevant for the current classification systems where the dominant arm is scored twice. It is recommended to restore this balance, based on an evidence-based weighting (14).

This study provides important insights into the role of trunk function and strength in WT mobility performance, although data should be interpreted with care. The sample consisted of elite international players, which strengthens the relevance for high-performance contexts, even if it limits generalizability to broader populations. Athlete inclusion was primarily determined by availability during major international tournaments, with an intentional focus on recruiting players with a broad range of trunk function levels. Although this resulted in a random sample of the elite WT population, the sample included a relatively large number of athletes with spinal cord injury and amputation, which may have influenced the observed relationships. As future work incorporates a larger number of athletes, it may become possible to explore subgroup differences within specific impairment groups. Female athletes were not represented in the Quad division; however, this reflects the current demographic, as less than 5% of the global Quad players are female. Data were collected on hardcourt surfaces, which are widely used in international competition, though surface type can influence WT performance (35). On surfaces with higher rolling resistance, such as clay or grass, trunk control may play an even greater role, as increased resistance typically requires larger stabilising and propulsion forces from the upper body. Future research should therefore examine whether the performance differences observed between trunk groups are amplified on these alternative surfaces. Trunk strength was estimated indirectly through functional scoring rather than direct measurement, yet this approach aligns with classification practice and offers practical feasibility. For future research, isolated and standardized trunk strength protocols similar to those used for arm strength would enhance precision. Moreover, WT performance is inherently multifactorial, shaped not only by physical capacity but also by technical and tactical elements. Integrating tennis-specific performance data, for example through Hawk-Eye technology, could provide a more comprehensive understanding of how strength and mobility relate to match outcomes, including stroke execution and court coverage. Addressing these factors will help validate and extend the current findings, ensuring that classification systems reflect the complex nature of WT performance. For example, future work may combine IMU-derived mobility measures with tennis-specific performance indicators such as Hawk-Eye ball-tracking metrics or match-analytics data on stroke execution and court-coverage patterns. Such integration would provide a more complete picture of how physical capacity interacts with tactical and technical performance in wheelchair tennis.

Given the clear and consistent differences in IMU-derived mobility patterns across trunk groups, IMU-based metrics may in the future serve as an objective component in classification research—for example by identifying characteristic acceleration or rotational signatures for trunk 0, 1 and 2. Building on the present findings, future studies will further investigate IMU-based quantification of trunk function and explore how such data could be translated into practical tools that provide classifiers with additional objective information during the classification process.

## 5 Conclusion

Trunk function plays a critical role in wheelchair tennis (WT) mobility performance, particularly in tasks involving rotation and acceleration, yet it is underrepresented in the current classification system. Our findings show that reduced active trunk range of motion (ROM) markedly affects mobility, creating clear performance gaps between trunk function groups. In contrast, upper-body strength only modestly correlates with mobility outcomes. Measured arm force depends on trunk stabilization, but static conditions and backrest support reduce its impact, resulting in less prominent differences in push and pull forces between trunk groups.

When refining the classification system, greater emphasis should be placed on measuring the impact of impairment on the ability to perform essential, sport-specific activities. This requires objective tests conducted in realistic, tennis-related contexts—such as dynamic trunk movements during acceleration and rotation—rather than relying solely on static strength measures. Such an approach ensures classification reflects functional limitations without aiming to predict overall performance.

Under the current classification system, trunk function accounts for only 14% of the total score (0–2 points out of 14), despite its strong influence on tasks involving rotation and forward acceleration, areas not fully captured by existing criteria. These findings suggest that trunk function should carry greater weight and be assessed more gradually. A possible additional partial trunk score, similar to wheelchair rugby, could be implemented (16). While differences in arm strength across dominant/non-dominant sides and the influence of racket use were observed, multiplying the dominant arm score (0–4) by two gives it disproportionate influence on the total classification score (>50%).

Future studies should integrate mobility measures with tennis-specific skills (e.g., stroke execution and court coverage) to develop a comprehensive evidence base for classification. Addressing these factors will ensure that classification reflects the complex nature of WT performance and supports a fair and functionally relevant system.

## Data Availability

The datasets presented in this article are not readily available because of ethical and privacy considerations, as the data involve elite athletes and detailed performance measures that could lead to participant identification. Data are available from the corresponding author upon reasonable request and with approval from the relevant ethics committee. Requests to access the datasets should be directed to Rienk van der Slikke; r.m.a.vanderslikke-1@tudelft.nl.
