# Supplementary material for: Trunk function: the core of mobility performance in wheelchair tennis
Source: Front Sports Act Living. 2026 Mar 25;8:1783088. doi: 10.3389/fspor.2026.1783088 (PMC13057481; doi:10.3389/fspor.2026.1783088)
Supplement: Supplementary file 1 [file Table1.pdf]

Supplementary Material

Table 1 Overview of the impairment types per trunk group.

Impairment Category	Trunk 0	Trunk 1	Trunk 2
**Spinal cord injury (SCI)**	8 (C6-T10)	9 (C7-T12)	8 (C7 - L1)
**Limb amputations**			8
**Congenital limb deficiency / dysmelia / skeletal disorders**			8
**Neurological impairments (non-SCI)**		1	5
**Cerebral palsy (CP)**			2
**Orthopaedic conditions**			2
	8	10	33
