# Supplementary material for: Trunk function: the core of mobility performance in wheelchair tennis
Source: Front Sports Act Living. 2026 Mar 25;8:1783088. doi: 10.3389/fspor.2026.1783088 (PMC13057481; doi:10.3389/fspor.2026.1783088)
Supplement: Supplementary file 2 [file Table2.pdf]

Table 2 Measured forces in eight different positions. The forearm measurements were conducted with the elbow at 90° flexion. All measurements were performed on both the left and right sides.

Direction	Full functional	Shoulder/elbow	Shoulder	Elbow
**Flexion**	Push	Full arm	Upper arm	Forearm
**Extension**	Pull	Full arm	Upper arm	Forearm
