# Supplementary material for: Trunk function: the core of mobility performance in wheelchair tennis
Source: Front Sports Act Living. 2026 Mar 25;8:1783088. doi: 10.3389/fspor.2026.1783088 (PMC13057481; doi:10.3389/fspor.2026.1783088)
Supplement: Supplementary file 3 [file Table3.pdf]

Table 3 Force measurement Spearman's rho correlations (all significant $p<0.05$)

	Flex up arm D	Flex up arm ND	Ext up arm D	Ext up arm ND	Flex forearm 90 D	Flex forearm 90 ND	Ext forearm 90 D	Ext forearm 90 ND	Push D	Push ND	Pull D	Pull ND
Flex up arm D		0.93	0.70	0.72	0.76	0.67	0.80	0.67	0.68	0.63	0.62	0.56
Flex up arm ND	0.93		0.72	0.76	0.74	0.74	0.85	0.70	0.69	0.67	0.61	0.62
Ext up arm D	0.70	0.72		0.92	0.85	0.78	0.85	0.81	0.69	0.72	0.70	0.78
Ext up arm ND	0.72	0.76	0.92		0.77	0.84	0.88	0.85	0.71	0.73	0.69	0.79
Flex forearm 90 D	0.76	0.74	0.85	0.77		0.75	0.86	0.73	0.71	0.69	0.67	0.67
Flex forearm 90 ND	0.67	0.74	0.78	0.84	0.75		0.83	0.81	0.61	0.74	0.65	0.81
Ext forearm 90 D	0.80	0.85	0.85	0.88	0.86	0.83		0.82	0.72	0.76	0.60	0.69
Ext forearm 90 ND	0.67	0.70	0.81	0.85	0.73	0.81	0.82		0.66	0.69	0.55	0.74
Push D	0.68	0.69	0.69	0.71	0.71	0.61	0.72	0.66		0.86	0.69	0.70
Push ND	0.63	0.67	0.72	0.73	0.69	0.74	0.76	0.69	0.86		0.68	0.76
Pull D	0.62	0.61	0.70	0.69	0.67	0.65	0.60	0.55	0.69	0.68		0.83
Pull ND	0.56	0.62	0.78	0.79	0.67	0.81	0.69	0.74	0.70	0.76	0.83	
