# Supplementary material for: Trunk function: the core of mobility performance in wheelchair tennis
Source: Front Sports Act Living. 2026 Mar 25;8:1783088. doi: 10.3389/fspor.2026.1783088 (PMC13057481; doi:10.3389/fspor.2026.1783088)
Supplement: Supplementary file 4 [file Table4.pdf]

Table 4 Cohen's d effect sizes and confidence intervals

		Standardizer	Point Estimate	95% Confidence Interval		Standardizer	Point Estimate	95% Confidence Interval		Standardizer	Point Estimate	95% Confidence Interval	
		0-2		Low	Up	0-1		Low	Up	1-2		Low	Up
Force	Push Max [N]	97.6	-0.51	-1.29	0.27	81.3	-0.53	-1.47	0.43	103.1	-0.07	-0.78	0.64
	Pull Max [N]	106.2	-0.95	-1.74	-0.14	85.3	-1.08	-2.07	-0.07	110.3	-0.07	-0.78	0.63
Match	Max speed [m/s]	0.43	-1.98	-2.85	-1.08	0.47	-0.56	-1.50	0.40	0.43	-1.35	-2.10	-0.57
	Avg forward acc [m/s2]	0.16	-1.41	-2.23	-0.57	0.15	-1.44	-2.48	-0.37	0.18	-0.04	-0.75	0.67
	Max rotational speed [o/s]	49.44	-1.50	-2.34	-0.66	33.75	-1.43	-2.47	-0.36	52.13	-0.50	-1.21	0.22
	Avg rotational acc [o/s2]	17.85	-1.54	-2.38	-0.69	15.76	-1.36	-2.39	-0.30	19.06	-0.32	-1.03	0.39
	Acc push (>mean) [m/s2]	0.88	-1.66	-2.50	-0.79	0.82	-0.48	-1.41	0.47	0.85	-1.24	-1.99	-0.48
Sprint 20m	Max speed [m/s]	0.51	-1.71	-2.56	-0.84	1.12	-0.01	-0.93	0.90	0.83	-1.04	-1.75	-0.32
	Acc push (>mean) [m/s2]	1.02	-2.39	-3.32	-1.44	0.87	-1.25	-2.26	-0.21	1.08	-1.24	-1.99	-0.48
	Time 20m [s]	0.72	1.54	0.68	2.39	0.90	0.71	-0.29	1.68	0.79	0.60	-0.16	1.35
	Avg trunk angle [o]	12.77	1.70	0.82	2.56	16.48	0.12	-0.86	1.10	14.27	1.38	0.53	2.21
	Acc backward [m/s2]	5.28	1.30	0.47	2.13	3.65	0.59	-0.39	1.56	5.41	0.87	0.10	1.63
Sprint 12m	Max speed [m/s]	0.41	-1.90	-2.77	-1.01	0.45	-0.60	-1.53	0.34	0.42	-1.23	-1.95	-0.49
	Acc push (>mean) [m/s2]	0.93	-2.37	-3.30	-1.43	0.88	-0.87	-1.81	0.10	0.95	-1.52	-2.27	-0.76
	Time 10m [s]	0.35	1.96	1.06	2.83	0.45	0.50	-0.46	1.43	0.38	1.24	0.48	1.99
	Avg trunk angle [o]	12.58	2.29	1.25	3.30	8.84	1.87	0.55	3.13	11.78	1.04	0.22	1.85
	Acc backward [m/s2]	4.08	1.77	0.89	2.64	4.33	0.73	-0.25	1.68	4.24	0.96	0.21	1.70
Sprint Interval	Max speed [m/s]	0.36	-2.02	-2.90	-1.11	0.42	-0.75	-1.68	0.20	0.37	-1.13	-1.85	-0.40
	Acc push (>mean) [m/s2]	0.81	-2.43	-3.36	-1.48	0.85	-0.76	-1.70	0.19	0.84	-1.57	-2.32	-0.80
	Time 10m [s]	0.84	1.71	0.83	2.56	0.92	1.16	0.15	2.13	0.78	0.48	-0.22	1.17
	Avg trunk angle [o]	13.03	1.66	0.78	2.52	11.79	0.97	-0.09	1.99	12.55	0.82	0.01	1.62
	Acc backward [m/s2]	3.09	1.61	0.75	2.47	3.51	0.41	-0.54	1.34	3.00	1.19	0.42	1.94
Sprint slalom	Max speed [m/s]	0.41	-1.88	-2.75	-0.99	0.50	-0.52	-1.44	0.41	0.44	-1.17	-1.89	-0.43
	Avg rotational speed [o/s]	4.90	-2.19	-3.09	-1.26	5.36	-0.93	-1.88	0.04	5.10	-1.12	-1.83	-0.39
	Acc push (>mean) [m/s2]	0.88	-2.36	-3.28	-1.41	0.95	-0.87	-1.81	0.10	0.93	-1.34	-2.07	-0.59
	Avg rotational acc [o/s2]	84.25	-1.18	-1.99	-0.36	76.59	-0.44	-1.35	0.49	88.34	-0.75	-1.45	-0.05
	Avg trunk angle [o]	11.59	2.32	1.31	3.31	8.86	1.66	0.40	2.87	10.97	1.11	0.23	1.97
	Acc backward [m/s2]	2.91	1.67	0.79	2.53	2.95	0.93	-0.06	1.90	2.69	0.78	0.04	1.51
Turn R	Max rotational speed [o/s]	58.38	-2.15	-3.05	-1.23	54.38	-1.16	-2.13	-0.15	60.84	-1.03	-1.74	-0.31
	Avg rotational acc [o/s2]	159.8	-1.97	-2.85	-1.07	130.1	-1.05	-2.01	-0.06	153.6	-1.16	-1.88	-0.43
	Avg trunk angle [o]	10.06	2.27	1.32	3.19	8.87	1.33	0.25	2.38	8.74	1.25	0.45	2.04
Turn L	Max rotational speed [o/s]	64.89	-1.79	-2.65	-0.91	57.78	-0.87	-1.82	0.09	64.92	-1.01	-1.72	-0.29
	Avg rotational acc [o/s2]	156.6	-1.67	-2.51	-0.80	123.9	-0.82	-1.76	0.14	148.8	-1.07	-1.79	-0.35
	Avg trunk angle [o]	11.01	1.99	1.07	2.88	10.71	1.24	0.17	2.27	9.60	0.90	0.12	1.67
