# Supplementary material for: Trunk function: the core of mobility performance in wheelchair tennis
Source: Front Sports Act Living. 2026 Mar 25;8:1783088. doi: 10.3389/fspor.2026.1783088 (PMC13057481; doi:10.3389/fspor.2026.1783088)
Supplement: Supplementary file 5 [file Table5.pdf]

Table 5 Force correlations (Pearson) with WMP outcomes in Match and on-court standardized tests. Only significant (p<0.05) correlations are shown. Note: All correlations are presented as positive for interpretability (e.g., shorter sprint times correspond to higher force)

**Pearson Correlation**		Flex_up_arm_D	Flex_up_arm_ND	Ext_up_arm_D	Ext_up_arm_ND	Flex_fore_arm_90_D	Flex_fore_arm_90_ND	Ext_fore_arm_90_D	Ext_fore_arm_90_ND	Push_D	Push_ND	Pull_D	Pull_ND	Push Max	Pull Max
Match	MaxSpeed				0.37					0.34	0.30	0.29	0.32	0.33	0.32
	Avg_acc			0.39	0.38		0.33			0.39		0.39	0.44	0.29	0.43
	MaxRoSpeed			0.36	0.34					0.35		0.35	0.34	0.30	0.36
	Avg_ro_acc				0.28					0.43		0.32	0.37	0.35	0.36
	Acc_push_om2_best5			0.33	0.35				0.32	0.32	0.38	0.34	0.42	0.37	0.40
Sprint 20m	MaxSpeed			0.43	0.43		0.32			0.35	0.32	0.37	0.40	0.34	0.40
	Acc_push_om2			0.48	0.39	0.42	0.42	0.43	0.44	0.38	0.38	0.47	0.50	0.39	0.50
	Time_20m			0.50	0.51	0.52	0.42	0.46	0.50	0.59	0.48	0.54	0.62	0.54	0.60
	Avg_trunk_ang														
	Backw_acc_pp														
Sprint 12m	MaxSpeed			0.52	0.52	0.39	0.41	0.41	0.43	0.51	0.50	0.57	0.61	0.53	0.61
	Acc_push_om2			0.46	0.40	0.31	0.34	0.36	0.35	0.34	0.38	0.43	0.43	0.38	0.44
	Time_10m			0.48	0.51	0.42	0.37	0.41	0.48	0.51	0.47	0.49	0.59	0.50	0.57
	Avg_trunk_ang			0.38	0.37				0.35				0.38		0.34
	Backw_acc_pp												0.31		0.29
Sprint Interval	MaxSpeed			0.50	0.52	0.35	0.38	0.38	0.40	0.49	0.51	0.52	0.58	0.54	0.57
	Acc_push_om2			0.53	0.46	0.36	0.35	0.39	0.39	0.42	0.42	0.43	0.46	0.45	0.47
	Time_10m	0.31	0.35	0.48	0.53	0.41	0.47	0.42	0.50	0.49	0.43	0.53	0.56	0.48	0.57
	Avg_trunk_ang			0.39	0.33		0.30		0.31				0.30		
	Backw_acc_pp			0.32	0.29			0.32					0.30		
Sprint slalom	MaxSpeed			0.44	0.46	0.35	0.32	0.36	0.40	0.47	0.44	0.47	0.53	0.47	0.52
	MeanRoSpeed			0.45	0.54	0.39	0.43	0.39	0.43	0.48	0.43	0.49	0.58	0.46	0.56
	Acc_push_om2			0.45	0.43	0.33	0.38	0.35	0.39	0.38	0.38	0.46	0.46	0.40	0.47
	Avg_ro_acc			0.41	0.42	0.32	0.38	0.35	0.32	0.40	0.45	0.40	0.52	0.46	0.48
	Avg_trunk_ang			0.31	0.32				0.34				0.34		0.31
	Backw_acc_pp			0.33	0.35		0.29	0.34			0.29	0.38	0.44		0.43
Turn R	MaxRoSpeed			0.45	0.52	0.36	0.36	0.34	0.41	0.51	0.41	0.56	0.61	0.47	0.61
	Avg_ro_acc			0.49	0.55	0.39	0.40	0.37	0.43	0.50	0.44	0.57	0.56	0.48	0.58
	Avg_trunk_ang														
Turn L	MaxRoSpeed			0.48	0.55	0.38	0.43	0.38	0.42	0.52	0.51	0.55	0.63	0.54	0.62
	Avg_ro_acc			0.37	0.50	0.34	0.31	0.32	0.38	0.46	0.43	0.47	0.48	0.47	0.49
	Avg_trunk_ang														
